# College Note

**Published:** 1901-11

**Authors:** 


### COLLEGE NOTE.

The University of Buffalo began its fifty-sixth regular collegiate session September 30, 1901, the opening lecture being delivered by Dr. F. C. Busch, professor of physiology. There was a large attendance of students and the college year made an auspicious beginning. Professor Busch reviewed the progress of medicine and surgery during the past century and spoke of the usefulness of the University of Buffalo in contributing to the general stock of medical learning. His address was well received.

